# A Choline Oxidase Amperometric Bioassay for the Detection of Mustard Agents Based on Screen-Printed Electrodes Modified with Prussian Blue Nanoparticles

**DOI:** 10.3390/s150204353

**Published:** 2015-02-13

**Authors:** Fabiana Arduini, Viviana Scognamiglio, Corrado Covaia, Aziz Amine, Danila Moscone, Giuseppe Palleschi

**Affiliations:** 1 Dipartimento di Scienze e Tecnologie Chimiche, Università di Roma Tor Vergata, Via Della Ricerca Scientifica, 00133 Rome, Italy; E-Mails: corrado.covaia@libero.it (C.C.); moscone@uniroma2.it (D.M.); palleschi@uniroma2.it (G.P.); 2 Dipartimento Agroalimentare, IC-CNR Istituto di Cristallografia, Via Salaria Km 29.3, 00015 Monterotondo Scalo, Rome 00015, Italy; E-Mail: viviana.scognamiglio@mlib.ic.cnr.it; 3 Laboratoire Génie des Procédés et Environnement, Faculté de Sciences et Techniques, Université Hassan II—Mohammedia, B.P.146, Mohammadia 20850, Morocco; E-Mail: azizamine@yahoo.fr

**Keywords:** mustard agents, inhibition, choline oxidase, screen-printed electrode, Prussian Blue nanoparticles

## Abstract

In this work a novel bioassay for mustard agent detection was proposed. The bioassay is based on the capability of these compounds to inhibit the enzyme choline oxidase. The enzymatic activity, which is correlated to the mustard agents, was electrochemically monitored measuring the enzymatic product, hydrogen peroxide, by means of a screen-printed electrode modified with Prussian Blue nanoparticles. Prussian Blue nanoparticles are able to electrocatalyse the hydrogen peroxide concentration reduction at low applied potential (−50 mV *vs.* Ag/AgCl), thus allowing the detection of the mustard agents with no electrochemical interferences. The suitability of this novel bioassay was tested with the nitrogen mustard simulant bis(2-chloroethyl)amine and the sulfur mustard simulants 2-chloroethyl ethyl sulfide and 2-chloroethyl phenyl sulfide. The bioassay proposed in this work allowed the detection of mustard agent simulants with good sensitivity and fast response, which are excellent premises for the development of a miniaturised sensor well suited for an alarm system in case of terrorist attacks.

## 1. Introduction

**Scheme 1.**
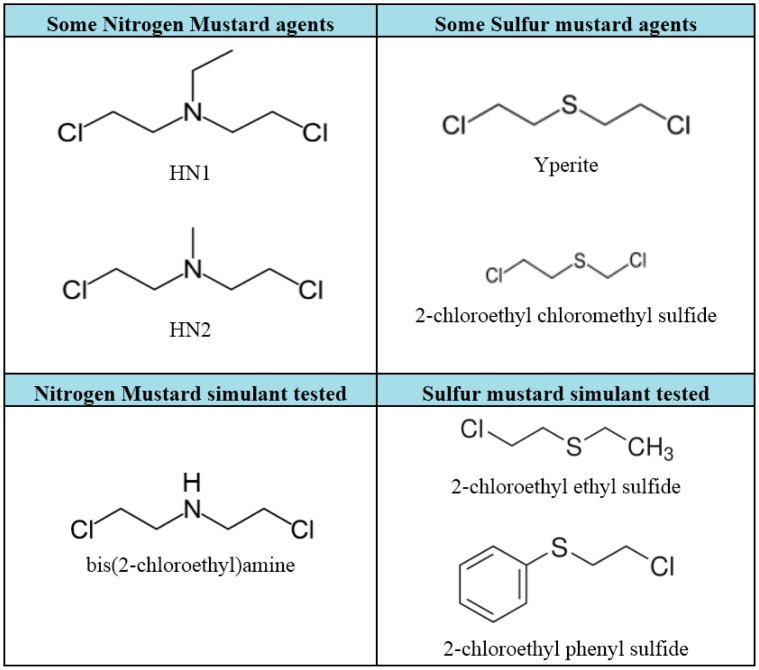
Mustard agents.

Chemical warfare agents (CWAs) have been deliberately produced and employed in the battlefields during the 20th century with the purpose of killing or debilitating living organisms. CWA are in all probability the cruellest produced among the Weapons of Mass Destruction (WMD). The Chemical Weapons Convention (CWC) has classified them based on their volatility, chemical structure or the physiological effects produced on humans. Regarding the latter feature, CWAs comprise nerve agents, vesicants (blistering agents), bloods agents (cyanogenic agents), choking agents (pulmonary agents), riot-control agents (tear gases), psychomimetic agents, and toxins [1]. Among them, vesicants are compounds able to generate toxic effects on living organisms and persistently contaminate soils and water, and comprise mustard agents (MAs) and arsenicals. MAs include sulfur mustards, such as Yperite (bis(2-chloroethyl)sulfide), 2-chloroethyl chloromethyl sulfide, and nitrogen mustards, such as HN1 (bis(2-chloroethyl)ethylamine), HN2 (bis(2-chloroethyl)methylamine) (Scheme 1), and the organic arsenical Lewisite (dichloro(2-chlorovinyl)-arsine). The name mustard agents arises from impure weapons-grade material, which has an odour similar to that of mustard or garlic [2].

Since 1993, CWC has promulgated several regulations, with new implementations since 1997, to prohibit the production and the use of chemical weapons, including mustard agents. Nevertheless, MAs and their degradation products, represent nowadays one of the most toxic environmental pollutants, being persistent in the environment for long term and causing high toxicity on biota and humans. MA exposure may occur through the skin, respiratory system, genital tract, ocular surface and gastrointestinal system, with serious acute and long term complications [3]. MAs are also known as DNA alkylating agents able to cause cytotoxicity, mitosis inhibition, mutagenesis, carcinogenesis, and colinomimetic effects. These mechanisms lead to final DNA damage, oxidative stress, impaired energy metabolism and consequently necrosis and cell death [4]. Several studies on battlefield victims have demonstrated that exposure to mustards is a traumatic event having long-lasting effects on mental health [5].

For these reasons, MAs remain some of the agents of highest internationally concern and are receiving increasing attention regarding their decontamination and degradation, but in particular for their detection in water and food or in human biological fluids. Gas chromatography (GC) and liquid chromatography (LC), combined with mass spectrometry (MS) are the main techniques employed for the screening of MAs in environmental samples [6] or in biological fluids [7]. Although they provide good sensitivity and powerful analytical potential, these techniques are complex, expensive, time-consuming, require qualified personnel, and are inadequate to operate in the field for screening analysis. Thus, due to the high number of exposures to MAs through different sources (e.g., recent terrorist attacks or already contaminated sites) and their chemical broad spectrum, there is an increasing interest in the development of highly sensitive, selective, contactless and early detection systems for low concentrations below the median lethal dose (LD_50_: dose required to kill half the members of a tested population).

In this context, biosensor technology has a great potential to address these challenges, through the development of tailor-made, fast and cost effective, small and portable instruments [8–12], with adequate sensitivity and selectivity, to unambiguously identify MAs. Several biosensors have been designed and realised for the detection of mustards based on chlorophyll fluorescence [13], whole cell Raman spectroscopy [14], molecular imprinting polymers [15], whole cell luminescence [16], or piezoelectric immunosensors [17]. Some analytical approaches present several disadvantages, such as weak operational stability, limited storage capacity, long response times, and laboratory set-up.

In the present work, we propose a new analytical device able to deal with the drawbacks related to sensitivity, time response, and portability. In particular, we developed an amperometric bioassay to reveal sulfur and nitrogen mustards based on their capability to inhibit the enzyme choline oxidase. This inhibitory effect was demonstrated in the work of Barron *et al.* in 1948, focused on the effects of mustards on the structural properties and chemical activity of different enzymes involved in metabolic processes. Among these enzymes, choline oxidase has been indicated as the most sensitive to the action of nitrogen mustards [18].

Thus, choline oxidase from *Alcaligenes* sp. was chosen as biological recognition element for the development of the bioassay, and the simulants nitrogen mustard bis(2-chloroethyl)amine and sulfur mustards 2-chloroethyl ethyl sulfide and 2-chloroethyl phenyl sulfide (Scheme 1) were selected as target analytes and amperometrically detected by following the enzymatic product hydrogen peroxide.

## 2. Experimental Section

### 2.1. Apparatus and Reagents

All chemicals from commercial sources were of analytical grade. Choline oxidase (EC 1.1.3.17) from *Alcaligenes* sp. and its substrate choline, potassium hydrogen phosphate, hydrogen peroxide, the nitrogen mustard simulant bis(2-chloroethyl) amine and the sulfur mustard simulants 2-chloroethyl ethyl sulfide and 2-chloroethyl phenyl sulfide were purchased from Sigma Chemical Co. (St. Louis, MO, USA). Potassium chloride, potassium ferricyanide and iron chloride were obtained from Carlo Erba (Milan, Italy). A PalmSens3 (Houten, The Netherlands) Potentiostat was used for amperometric/voltammetric measurements.

### 2.2. Preparation of PBNPs-SPE

**Figure 1.**
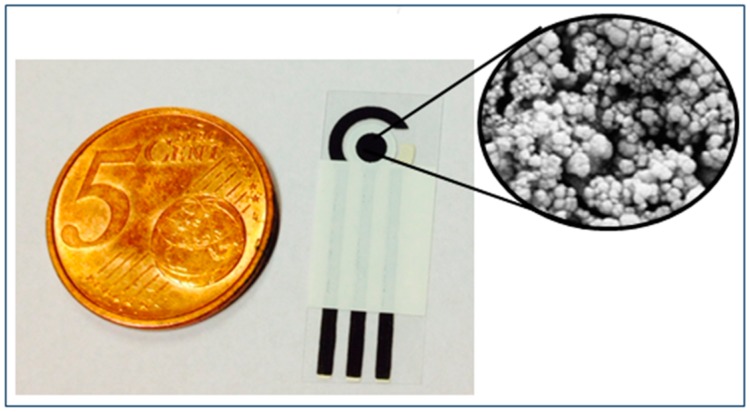
Photo of an SPE modified with PBNPs. Inset: SEM imagine of the working electrode modified with PBNPs (PBNP diameter 95 ± 15 nm). The 5 cent euro coin reference has a diameter of 22.25 mm.

Screen-printed electrodes (SPEs) were produced in our laboratory by thick film technology (TFT) with a 245 DEK (Weymouth, UK) screen-printing machine. SPEs consisted of a working electrode in graphite modified with Prussian Blue nanoparticles (PBNPs), a reference electrode in silver/silver chloride, and a counter electrode in graphite (Figure 1). Graphite based ink (Electrodag 421, Acheson, Henkel, UK), silver/silver chloride ink (Electrodag 4038 SS) and insulating ink (Carboflex 25.101.S, Acheson, Henkel, UK) were used. A flexible polyester film (Autostat HT5) obtained from Autotype Italia (Milan, Italy) was used as substrate. The electrodes were home produced in sheets of 48 each. The diameter of the working electrode was 0.3 cm, resulting in a geometric area of 0.07 cm^2^ [19]. The SPEs were then modified with Prussian Blue nanoparticles and this was accomplished by placing a drop (10 μL total volume) of “precursor solution” on the working electrode area [20]. This solution was obtained by mixing 5 μL of 0.1 M potassium ferricyanide in 10 mM HCl with 5 μL of 0.1 M ferric chloride in 10 mM HCl directly on the surface of the working electrode. The drop was carefully pipetted to be localized exclusively on the working electrode area. The solution was left on the electrode for 10 min and then rinsed with a few mL of 10 mM HCl. The electrodes were then left 90 min in the oven at 100 °C. The PBNP-modified electrodes were stored dry at room temperature in the dark.

### 2.3. Instrumentation and Bioassay Configuration

The amperometric measurements were performed exploiting PBNPs-SPEs as electrochemical sensor connected to a potentiostat (PalmSens3 Potentiostat) able to apply the adequate potential and send to a computer the registered response signals. 100 μL of a solution containing 0.05 M phosphate buffer + 0.1 M KCl, pH 7.4 was drop-casted on the sensor surface being attention to cover all three electrodes (working, reference and counter electrode). A potential of −50 mV *vs.* Ag/AgCl was applied and a signal current was registered. The electrode surface was washed with distilled water. Then, 100 μL of a solution containing 0.05 M phosphate buffer + 0.1 M KCl pH 7.4, 180 mU/mL choline oxidase and 0.5 mM choline was drop-casted on the electrode surface and a signal current was registered for a period of 3 min to follow the current signal increasing, corresponding to the enzymatic reaction progress. The final current signal corresponds to A_0_. The electrode surface was thus washed with distilled water once more. To quantify the inhibitor, 70 μL of a solution containing 0.05 M phosphate buffer + 0.1 M KCl, pH 7.4 and 180 mU/mL choline oxidase was incubated for 20 s with 10 μL of the inhibitor to a final concentrations ranging from 0 to 2.5 mM and drop-casted to the electrode surface. After incubation 20 μL of choline for a final concentration of 0.5 mM was added and a signal current increasing was registered for 3 min, corresponding to the enzymatic reaction progress. The final current signal corresponds to A_1_. Then, the inhibition percent was calculated according to the equation:

(1) $$\text{I}\%=\left[\left({\text{A}}_{0}-{\text{A}}_{1}\right)/{\text{A}}_{0}\cdot 100\right]$$

where A_0_ is enzyme activity in the absence of inhibitor, A_1_ is enzyme activity in presence of inhibitor.

### 2.4. Safety Conditions

Mustard simulant stock solutions were prepared under appropriate safety conditions. Operators were dressed with lab dresses, gloves, mask, and glasses to avoid contact or inhalation with powder and/or vapour. In addition, a dedicated hood has been used during sample preparation and analysis.

## 3. Results and Discussion

The amperometric bioassay was set up by exploiting choline oxidase (CO) from *Alcaligenes* sp. as biological recognition element for the detection of nitrogen and sulfur mustard agent simulants: bis(2-chloroethyl)amine, 2-chloroethyl ethyl sulfide and 2-chloroethyl phenyl sulfide. Several parameters were optimised to highlight the operational conditions of the analysis, including enzyme concentration, substrate concentration, and incubation time between the enzyme and simulant. The type of inhibition was further investigated to highlight if the mechanism was reversible or irreversible. Reversible inhibition is characterised by non-covalent interactions between inhibitor and enzyme with the consequent restoration of the initial activity after the inhibition measurements. On the contrary, in the case of irreversible inhibition, characterised by covalent bonding between the enzyme and the inhibitor, the restoration of the initial activity requires a reactivation of the enzyme using specific compounds. From an analytical point of view, these mechanisms should be clarified since the reversible inhibition does not require incubation time and the measurement is then characterised by a short time of analysis. On the other hand, extended incubation times are necessary in the case of irreversible inhibition to enhance the sensitivity.

### 3.1. Bioassay Configuration

Choline oxidase belongs to the family of oxidoreductase enzymes and catalyses the hydrolysis of acetylcholine. The enzymatic reaction produces hydrogen peroxide (H_2_O_2_) as a by-product, an electroactive molecule successively reduced on PBNPs-SPEs, since PBNPs are able to electrocatalyse the reduction of H_2_O_2_ at an applied potential of −50 mV *vs.* Ag/AgCl [20]. The generated amperometric signals are linearly dependent on the H_2_O_2_ concentration produced by the enzyme. Through the kinetic study of the production of H_2_O_2_, the enzymatic activity can be monitored in the presence of possible inhibitors (*i.e.*, mustard agents or their simulants). These inhibitors were able to decrease the enzyme activity, with a consequently reduction of H_2_O_2_ concentration enzymatically produced. The reduction of H_2_O_2_ leaded to a decrease of the amperometric signal proportional to the inhibitor concentration.

### 3.2. Optimization of the Amount of Choline Oxidase and Choline

**Figure 2.**
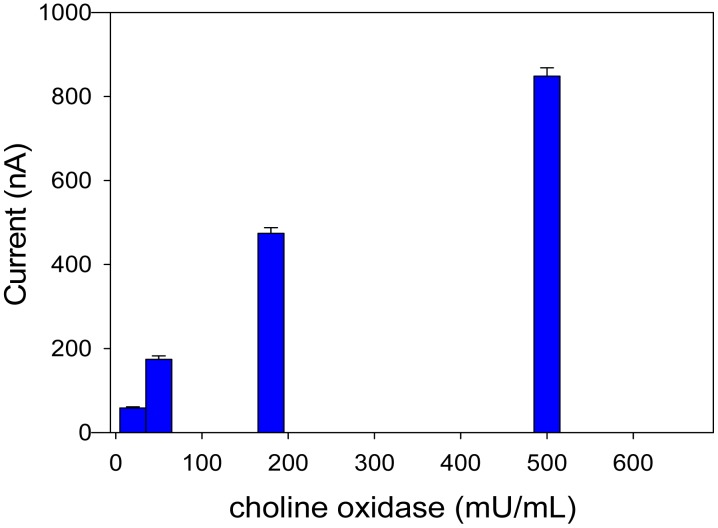
Bioassay response by varying the amount of choline oxidase. Choline at final concentration of 0.5 mM, applied potential: −50 mV *vs.* Ag/AgCl, 0.05 M phosphate buffer + 0.1 M KCl, pH 7.4. Measurements were carried out in triplicate.

In order to optimize the concentration of choline oxidase, a study on the enzyme response as a function of enzymatic units was carried out. According to the experimental data presented in Figure 2, a choline oxidase concentration of 180 mU/mL was selected. This choice represents a compromise between cost of the enzyme and measurement accuracy. For instance, using a lower enzyme concentration (*i.e.*, 20 mU/mL) current values of about 60 nA were obtained with a RSD of 4.3%; instead, using a higher enzyme concentration (*i.e.*, 180 mU/mL), the higher current values of about 500 nA were reached with a lower RSD of 2.9%. As expected, a lower repeatability was obtained using 3 min as reaction time with 20 mU/mL enzyme; indeed an extended reaction time was required to reach current intensities with higher accuracy. On the other hand, an enzyme concentration of 500 mU/mL allowed a lower RSD of 2.3%; however, the analysis was more expensive due to the cost of the additional enzyme.

**Figure 3.**
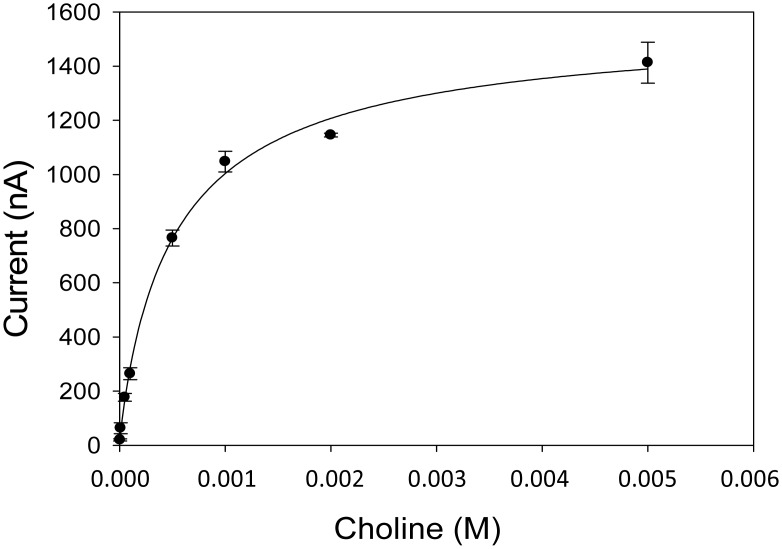
Choline calibration plot. Choline oxidase concentration: 180 mU/mL; applied potential: −50 mV *vs.* Ag/AgCl; 0.05 M phosphate buffer + 0.1 M KCl, pH 7.4. Measurements were carried out in triplicate.

Using an enzyme concentration of 180 mU/mL, a calibration curve for choline measurement was recorded (Figure 3). An increase of the reduction current occurred in the presence of substrate, and the current was registered and sampled at 180 s, showing amperometric signals proportional to H_2_O_2_ concentration and consequently to the reaction rate as a function of a substrate concentration increase. The Michaelis-Menten constant, K_M_, for choline was determined using the Michaelis-Menten equation:

(2) $$V_{0}=\frac{V\max\cdot S}{Km+S_{0}}$$

Thus resulting in a K_M_ of 0.47 ± 0.4 mM, which is, for instance, higher than the one found using the immobilised enzyme since usually in solution the enzyme has a better affinity for the substrate compared to the immobilised enzyme [21].

A final concentration of 0.5 mM choline was chosen for the measurements, since a higher concentration could compromise the identification of a competitive inhibition of reversible nature.

### 3.3. Inhibition Studies

**Figure 4.**
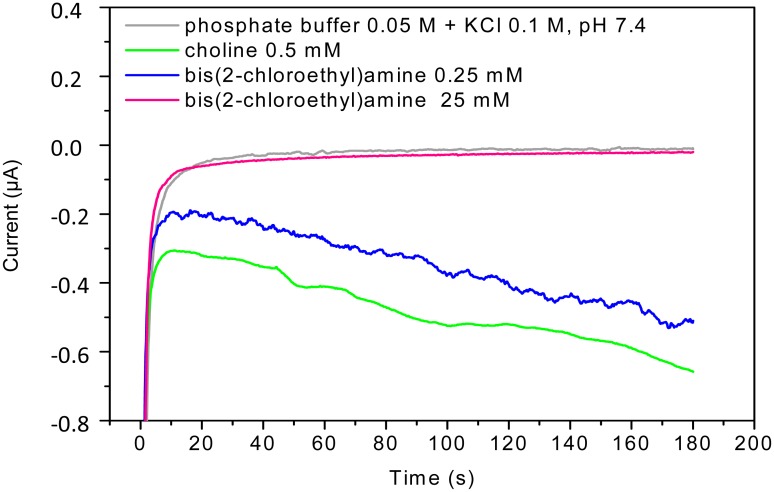
Original recording obtained using the bioassay*.* Applied potential −50 mV *vs.* Ag/AgCl; Choline oxidase concentration: 180 mU/mL. Signal recorded in 0.05 M phosphate buffer solution + 0.1 M KCl, pH 7.4 (grey line) and in a solution of choline (0.5 mM) in 0.05 M phosphate buffer solution + 0.1 M KCl, pH 7.4 in absence (green line) and in presence of bis(2-chloroethyl)amine 0.25 mM (blue line) and 25 mM (pink line).

The sulfur mustard agent simulants (2-chloroethyl ethyl sulfide and 2-chloroethyl phenyl sulfide, also known as half-mustards) and the nitrogen mustard agent simulant bis(2-chloroethyl)amine were used as target analytes. Since MAs are highly toxic and their use in no-security laboratories is restricted, the bioassay was developed using these simulants employed also in literature [22]. Figure 4 showed a typical amperogram indicating values of the current reduction in function of time, derived from the enzymatic production of H_2_O_2_. A decrease of the amperometric signals occurred upon increasing of the inhibitor concentration. In particular, the grey line represented an analysis of a solution of 0.05 M phosphate buffer + 0.1 M KCl, pH 7.4 in the absence of choline oxidase, where the signal was constant and close to zero; green line represented an analysis of a solution of 0.05 M phosphate buffer + 0.1 M KCl, pH 7.4 in the presence of choline oxidase and choline, where the signal indicated the production of H_2_O_2_ due to the enzymatic reaction; whereas blue and pink lines were signals of a solution of 0.05 M phosphate buffer + 0.1 M KCl, pH 7.4 in the presence of choline oxidase, choline and bis(2-chloroethyl)amine at a concentration of 0.25 mM and 25 mM, respectively. At high concentration of simulant (25 mM) the inhibition percentage was close to 100%.

The inhibition mechanism and its percentage was further investigated in function of the incubation time and enzymatic units (U/mL), with the aim of highlighting which kind of inhibition (reversible or irreversible) takes place between the enzyme and the sulfur or nitrogen mustard simulants. Since the reversible inhibition is not dependent by the abovementioned parameters, while the irreversible one is directly proportional to the incubation time, but inversely proportional to the enzyme units, the enzyme inhibition mechanism can be unequivocally demonstrated. Thus, studies on incubation time and enzyme units were provided for each simulant.

#### 3.3.1. Optimization of Incubation Time and Enzyme Concentration

##### Nitrogen Mustard Agent Simulants

**Figure 5.**
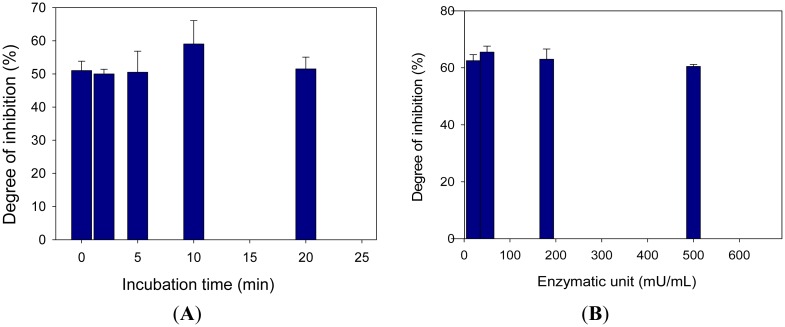
(**A**) Study of percentage inhibition *vs*. incubation time*.* Choline oxidase concentration: 180 mU/mL; choline concentration: 0.5 mM; bis(2-chloroethyl)amine concentration: 2.5 mM; phosphate buffer 0.05 M + 0.1 M KCl, pH 7.4; potential: −50 mV *vs.* Ag/AgCl; (**B**) Study of percentage inhibition *vs*. enzymatic units. Choline concentration: 0.5 mM; bis(2-chloroethyl)amine concentration: 2.5 mM; phosphate buffer 0.05 M + 0.1 M KCl, pH 7.4; potential: −50 mV *vs.* Ag/AgCl; incubation time: 120 s. Measurements were carried out in triplicate.

Analyses of the percentage of inhibition due to the simulant bis(2-chloroethyl)amine were investigated as a function of incubation time and enzyme units. As reported in Figure 5A,B, the results showed that the percentage of inhibition was almost constant in function of the two parameters, indicating that the inhibition mechanism was reversible, as well as the measurements of satisfactory reproducibility.

**Figure 6.**
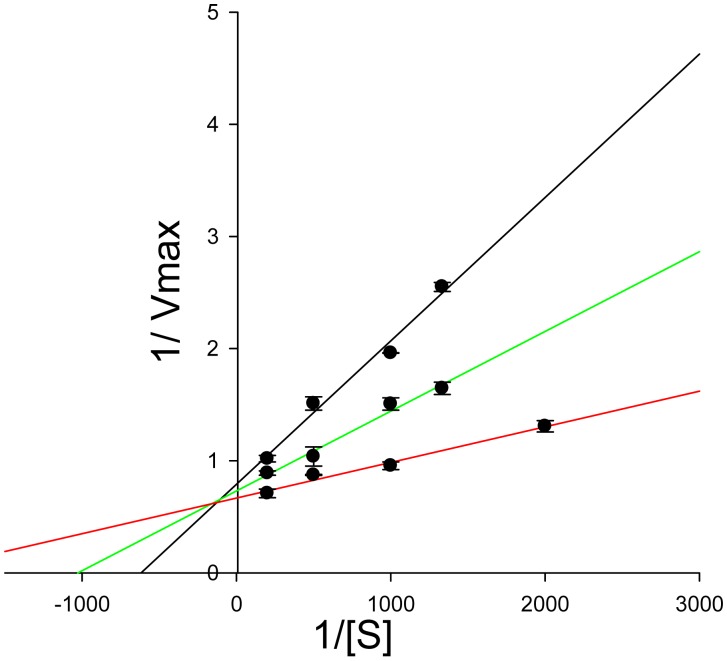
Lineweaver-Burk diagram for bis(2-chloroethyl)amine. Amperometric measurements of different concentrations of bis(2-chloroethyl)amine: plot a (black line): 2.5 mM; plot b (green line): 1 mM; plot c (red line): 0 mM. Choline oxidase concentration: 180 mU/mL; choline concentration: 0.5 mM; phosphate buffer 0.05 M + 0.1 M KCl, pH 7.4; potential: −50 mV *vs.* Ag/AgCl; incubation time: 120 s. Measurements were carried out in triplicate.

This reversible inhibition, described by means of Lineweaver-Burk diagram so called double reciprocal plot, a graphical representation of the equation of enzyme kinetics [23], was reported for bis(2-chloroethyl)amine in Figure 6. The results allowed highlighting the type of inhibition as mixed-competitive. Furthermore, a calibration curve of bis(2-chloroethyl)amine was constructed with a non-saturating substrate concentration. The obtained curve showed a linear range between 0.45 mM and 3 mM of the inhibitor described by the equation *y* = (10.8 ± 1.4)*x* + (19.5 ± 4.5) with a regression coefficient (R^2^) of 0.940, where *y* is the degree of inhibition and x is the concentration of inhibitor. A limit of detection of 0.45 mM corresponding to an inhibition of 20% was obtained. As expected, the IC_50_ found (2.8 mM) was much higher than the one (0.001 mM) reported using the mustard agent tris(β-chloroethyl)amine (HN3) [18].

##### Sulfur Mustard Agent Simulants

Inhibitory studies on the enzyme choline oxidase have been conducted with nitrogen mustards, as reported in [18]. In this work we provided inhibitory measurements also using sulfur mustards in order to assess whether the system was capable of measuring both types of inhibitor. Thus, the sulfur mustard agent simulants 2-chloroethyl ethyl sulfide and 2-chloroethyl phenyl sulfide were tested.

**Figure 7.**
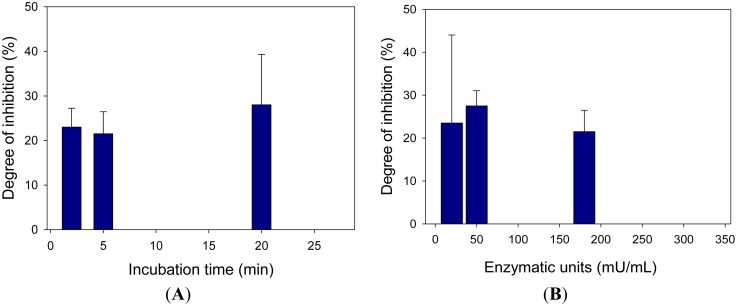
(**A**) Study of percentage inhibition *vs.* incubation time. Choline oxidase concentration: 180 mU/mL; choline concentration: 0.5 mM; 2-chloroethyl ethyl sulfide concentration: 0.01 mM; Phosphate buffer 0.05 M + 0.1 M KCl, pH 7.4; potential: −50 mV *vs.* Ag/AgCl; (**B**) Study of percentage inhibition *vs* enzymatic units. Choline concentration: 0.5 mM; 2-chloroethyl ethyl sulfide concentration: 0.01 mM; Phosphate buffer 0.05 M + 0.1 M KCl pH 7.4; potential: −50 mV *vs.* Ag/AgCl; incubation time: 120 s. Measurements were carried out in triplicate.

As previously described for bis(2-chloroethyl)amine, incubation time and enzyme units were investigated for the inhibition due to 2-chloroethyl ethyl sulfide, showing that the inhibition percent did not vary significantly thus indicating a reversible inhibition (Figure 7). Since a reversible type inhibition was observed, the incubation time of 120 s, previously chosen for nitrogen mustard simulant, was also selected for 2-chloroethyl ethyl sulfide.

Additionally, a calibration curve for 2-chloroethyl ethyl sulfide was constructed with a non-saturating substrate concentration. The obtained curve showed a linear range between 7 μM and 0.053 mM described by the equation *y* = (844 ± 66)*x* + (14 ± 3) with a regression coefficient (R^2^) of 0.967, with a limit of detection of 7 μM corresponding to an inhibition of 20%. In this case, a lower IC_50_ was found (0.04 mM), if compared with the previous one (2.8 mM) obtained using nitrogen mustard simulant. The limits of detection obtained using the proposed method were higher than standard laboratory set-up analyses (HPLC, GS-MS), by which very sensitive limit of detection were obtained (237 ng/g) [24]. However, our results can be considered competitive if compared with a disposable assay recently reported in literature using an optical detection which allows the detection limit of 50 μM and 10 μM by visual and fluorescence methods, respectively [25].

**Figure 8.**
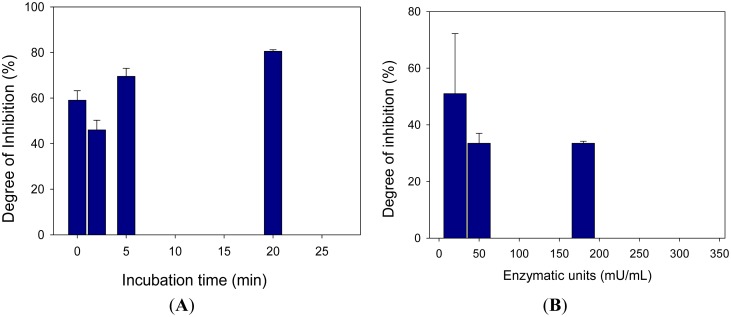
(**A**) Study of percentage inhibition *vs.* incubation time*.* Choline oxidase concentration: 180 mU/mL; choline concentration: 0.5 mM; 2-chloroethyl phenyl sulfide concentration: 0.68 mM; Phosphate buffer 0.05 M + 0.1 M KCl, pH 7.4; potential: −50 mV *vs.* Ag/AgCl; (**B**) Study of percentage inhibition *vs.* enzymatic units. Choline concentration: 0.5 mM; 2-chloroethyl phenyl sulfide concentration: 0.68 mM; Phosphate buffer 0.05 M + 0.1 M KCl, pH 7.4; potential: −50 mV *vs.* Ag/AgCl; incubation time: 120 s. Measurements were carried out in triplicate.

Incubation time and enzyme units were also investigated due to the inhibition by 2-chloroethyl phenyl sulfide (Figure 8). Results indicated a reversible inhibition also in this case. Moreover, a calibration curve for 2-chloroethyl phenyl sulfide was constructed with a non-saturating substrate concentration. The obtained curve described by non-linear four parameter logistic calibration plots showed a linear range between 0.3 mM and 1 mM of the inhibitor, a limit of detection of 0.1 mM corresponding to an inhibition of 10% and a IC_50_ equal to 0.7 mM. In this case the IC_50_ was higher than the one previously obtained using 2-chloroethyl phenyl sulfide as the sulfur mustard, which can probably be ascribed to the steric hindrance of the phenyl group. Furthermore, the best reproducibility was observed in the case of the nitrogen mustard simulant tested.

Taking into account the obtained results, a reversible mechanism was suggested for the analysed simulants, having advantages in terms of short analysis time and higher number of analyses using immobilised enzymes [26,27]. The obtained results have demonstrated the possibility of determining nitrogen and sulfur mustard agents using a portable and easy-to-use system based on the inhibition of choline oxidase, extending the few biochemical studies present in the literature on the inhibitory effect of mustards on choline oxidase only focused on nitrogen mustards [18,28,29].

## 4. Conclusions

In this work we have carried out preliminary studies on the inhibition of the enzyme choline oxidase due to mustard agent simulants, in order to develop a bioassay for the determination of the modeled mustard agent compounds in contaminated environmental sites, considering crucial features for tailor-made investigations such as short time response, simple equipment, reduced costs, and in-field analyses.

Based on the few biochemical studies reported in the literature on the inhibitory effect of mustards on choline oxidase [18,27,28], we performed further investigations on the mechanism of inhibition of both sulfur and nitrogen mustards (2-chloroethyl ethyl sulphide, 2-chloroethyl phenyl sulphide, bis(2-chloroethyl)amine) on the enzyme using a disposable and miniaturised electrochemical bioassay. Our results demonstrated that these compounds have the capability to reversibly inhibit the enzyme choline oxidase, and in the case of nitrogen mustard simulant (bis(2-chloroethyl)amine) a reversible mixed-competitive mechanism was suggested. Analytical parameters were also considered with the aim of developing a biosensing system for the qualitative and quantitative detection of mustards in the field. Data showed that analysis of these simulants can be achieved with a limit of detection of 0.45 mM for bis(2-chloroethyl)amine, 0.1 mM for 2-chloroethyl phenyl sulfide, and 7 μM for 2-chloroethyl ethyl sulfide. The analytical performances obtained make this bioassay a promising tool for rapid screening of mustard agents.
